# Novel anti-inflammatory drugs for the treatment of diabetic kidney disease

**DOI:** 10.1007/s00125-016-4030-4

**Published:** 2016-06-23

**Authors:** Hiddo J. L. Heerspink, Dick De Zeeuw

**Affiliations:** Department of Clinical Pharmacy and Pharmacology, University of Groningen, University Medical Center Groningen, PO Box 30001, 9700 RB Groningen, the Netherlands

**Keywords:** CCL2, Diabetic kidney disease, Inflammation, JAK-STAT, MCP-1

Over the last decade, numerous studies have demonstrated that inflammation is involved in the cascade of events that lead to renal complications in patients with diabetic kidney disease [1]. Experimental studies highlighted the pathogenic role of inflammatory processes at the cellular and molecular levels. Observational studies have translated these findings to the human situation and have consistently shown that diabetic patients with higher levels of inflammatory markers in their blood or urine are at higher risk of renal function decline. These studies raise the possibility that anti-inflammatory interventions may slow the progression of diabetic kidney disease.

The question of whether anti-inflammatory interventions protect the kidney is not new—the potential renoprotective effects of non-steroidal anti-inflammatory drugs (NSAIDs) were assessed in patients with proteinuria more than 30 years ago. An observational study of patients with nephropathy and proteinuria found that the incidence of doubling of serum creatinine or chronic dialysis was significantly lower among patients receiving indometacin [2]. These studies hypothesised that treatment with NSAIDs, such as indometacin or naproxen, confer renoprotection by reducing intra-glomerular pressure through haemodynamic effects [3, 4]. Whether anti-inflammatory properties contributed to the alleged long-term renoprotective effects of NSAIDs could not be determined at the time the studies were performed because specific assays for inflammatory markers were not available.

In recent years various novel inflammatory components have been discovered. These inflammatory components often interact and collectively form a complex interplay through which inflammation contributes to the development and progression of diabetic kidney disease [1]. The discovery of these inflammatory components has spurred scientists to develop new drugs targeting them. At the scientific meeting of the EASD in September 2015 in Stockholm three Phase 2 studies on novel anti-inflammatory agents were presented at a symposium entitled ‘Anti-inflammatory interventions in diabetes’. The first study investigated the effect of emapticap pegol (NOX-E36). Emapticap is a direct inhibitor of monocyte chemotactic protein-1 (MCP-1, also referred to as C-C motif chemokine ligand 2 or CCL2). MCP-1 is a chemokine produced by many cell types, including endothelial and epithelial cells as well as smooth muscle cells. Binding of MCP-1 to its receptor, C-C motif chemokine receptor 2 (CCR2), stimulates the release of monocytes from bone marrow and activates the migration and translocation of monocytes and macrophages [5]. Blockade of MCP-1 may alleviate a proinflammatory state and it is thought that intra-renal blockade of MCP-1 may preserve podocyte numbers as well as renal function. The Phase 2a study presented by Hermann Haller at the EASD, and recently published [6], was a randomised placebo-controlled double-blind trial in 75 patients with type 2 diabetes and micro- or macroalbuminuria. Emapticap or matched placebo were administered subcutaneously twice weekly for a total duration of 12 weeks. After 12 weeks’ treatment, emapticap had non-significantly decreased albuminuria by 15% (*p* = 0.22) [6]. Interestingly, the effects of emapticap on albuminuria persisted during the 12-week observational study period following the cessation of study medication [6].

The second study, presented by Dick de Zeeuw, also used a drug that targeted the MCP-1 axis. The study tested the albuminuria-lowering effect of the CCR2 antagonist CCX140-B in patients with type 2 diabetes and macroalbuminuria. As reported previously [7], 12 weeks’ treatment with CCX140-B at doses of 5 mg/day decreased albuminuria by 18% relative to placebo. The albuminuria-lowering effects persisted throughout the 52-week follow-up period, and 4 weeks after study drug discontinuation. Intriguingly, CCX140-B at doses of 10 mg/day also decreased albuminuria during the first weeks of treatment, but the effect dissipated during the 52-week follow-up period. In the 10 mg arm, a statistically significant increase in MCP-1, the endogenous ligand for CCR2, was also observed at the end of the 52-week follow-up. Interestingly, a post hoc analysis revealed that those patients with a higher increase in MCP-1 after 52 weeks showed a smaller decrease in albuminuria response. These findings raise the possibility that the higher increase in MCP-1 in the 10 mg arm competed with CCX140-B for binding to the receptor and blunted the albuminuria-lowering efficacy. With respect to safety, CCX140-B was generally well tolerated.

The results for a third drug were presented by Frank Brosius, who reported on baricitinib, a Janus kinase 1/2 (JAK1/2) inhibitor that is currently being tested in Phase 3 clinical trials for rheumatoid arthritis. In the last couple of years it has become increasingly clear that the JAK–signal transducers and activators of transcription (STAT) pathway is involved in transmitting responses of cytokines and chemokines to the nucleus in order to activate a range of cellular responses, thereby mediating a proinflammatory milieu [8]. As described in the review by Brosius et al in this issue of *Diabetologia* [9], treatment with baricitinib at doses ranging from 0.75 to 4 mg dose-dependently decreased albuminuria. The effects were present after 3 months’ treatment and persisted after 6 months. Anaemia was the most common reported side effect and occurred mainly with the 4 mg baricitinib dose.

Although these studies provide some indication that anti-inflammatory strategies targeting the MCP-1/CCL2 or the JAK–STAT pathway may be a fruitful approach to delay the progression of diabetic kidney disease, many questions remain unanswered. The first question that should be addressed is whether a change in albuminuria is the right surrogate measure to determine the efficacy of anti-inflammatory drugs. Although albuminuria is often used in Phase 2 clinical trials of diabetic kidney disease to assess drug efficacy, it may be possible that other more specific inflammatory markers are better surrogates to determine efficacy. However, to the best of our knowledge there are no data to prove that a drug-induced reduction in inflammatory markers is associated with a reduction in hard renal endpoints. In addition, it should be noted that, in theory, anti-inflammatory drugs might not decrease albuminuria but may still be renoprotective. It has been shown that albumin re-uptake at the tubular level triggers toxic effects and inflammatory responses [10]. This could mean that blocking proinflammatory pathways downstream of albumin re-uptake may prevent renal function loss without affecting albuminuria itself. Clearly, further research is required to assess whether albuminuria is the right surrogate biomarker for these agents and whether more specific inflammatory biomarkers could be better substitutes for end-stage renal disease.

Another question to be answered relates to the time course of the anti-inflammatory and albuminuria-lowering effects. MCP-1 inhibition with emapticap did not show an effect on albuminuria during 12 weeks’ treatment relative to placebo, but albuminuria levels tended to be lower during the 12 week period after drug discontinuation compared with placebo. In contrast, blockade of CCR2 (the endogenous receptor of MCP-1) with CCX140-B decreased albuminuria after 12 weeks’ treatment and the effect was already present after 4 weeks’ treatment. The discrepancy between these different time courses of effects is unclear and may relate to the different pharmacochemical and physical properties of the drugs, or could even be a chance finding given the low number of patients in these studies and the high variability in albuminuria in the placebo arms. A better understanding of the time course of effect, particularly for emapticap, is required before large outcome studies are initiated.

A third question that should be addressed is whether the anti-inflammatory agents exert structural effects and improve underlying disease pathophysiology. In all three studies the albuminuria-lowering effect persisted after study drug discontinuation. Specifically, even after 12 weeks’ discontinuation of emapticap, albuminuria did not return to baseline levels. These long-lasting effects suggest that the anti-inflammatory drugs may improve underlying pathophysiology. However, the 4-week follow-up period after cessation of treatment in the CCX140-B and baricitinib studies was too short to characterise long-lasting effects. Additional data supporting potential structural improvements in kidney pathology by these agents, for example, through imaging studies, are required and would also improve our understanding of the mechanisms of action of these anti-inflammatory agents.

How do we move on from here? Diabetic kidney disease remains associated with a high residual risk, and novel treatments to improve the prognosis of these high risk patients are highly desirable. The studies presented at the EASD meeting in Stockholm provide some hope that in the future novel treatments that employ other pathways beyond the well-known renin–angiotensin system may become available. However, there are still many questions to be answered, the most important of which is probably: how effective are these anti-inflammatory agents at preventing end-stage renal disease?

## Abbreviations

CCL2: C-C motif chemokine ligand 2
CCR2: C-C motif chemokine receptor 2
MCP-1: Monocyte chemotactic protein-1
NSAIDs: Non-steroidal anti-inflammatory drugs
